# Current Status of Macronutrient and Energy Intake and Metabolism Among High-Altitude Populations: A Systematic Review

**DOI:** 10.3390/nu18040572

**Published:** 2026-02-09

**Authors:** Yiyan Huang, Bin Li, Li Wang, Xueni Fan, Macairenzhuoma, Meng Zhang, Wenfeng Wang, Xiaodan Huang

**Affiliations:** 1 School of Public Health, Lanzhou University, Lanzhou 730000, China; hyiyan2024@lzu.edu.cn (Y.H.); fanxn2024@lzu.edu.cn (X.F.); 2 Xizang Academy of Agricultural and Animal Husbandry Sciences, Lhasa 850000, China; xukesuolibin@163.com (B.L.); wangli@taaas.org (L.W.); zhuoma98765@163.com (M.); zmtibet@163.com (M.Z.)

**Keywords:** high-altitude populations, macronutrient intake, energy intake, dietary patterns, nutritional status

## Abstract

**Background/Objectives:** Residents of high-altitude regions are chronically exposed to hypoxic and cold environments, which may alter energy metabolism and nutritional requirements, leading to distinct macronutrient intake patterns. However, existing evidence remains fragmented. This systematic review aimed to summarize macronutrient and energy intake among high-altitude populations and compare reported intake levels with dietary reference values to identify potential nutritional risks. **Methods:** A systematic search of PubMed and Web of Science was conducted for studies published between January 1990 and March 2025. Observational studies reporting dietary intake or dietary patterns among populations living at altitudes of ≥1500 m were eligible. Two reviewers independently screened studies, extracted data, assessed study quality using standardized tools, and synthesized results narratively. Study quality was assessed using the Quality Assessment Tool for Studies with Diverse Designs (QATSDD), with studies scoring ≥60% of the maximum score considered to indicate moderate or higher methodological quality. This review was not registered. **Results:** A total of 14 studies were included. Energy intake was generally within or close to recommended levels, with suboptimal intake observed in specific regions and population groups. Carbohydrate intake remained high, reflecting grain-based diets, while protein intake usually met or exceeded recommendations. Fat intake showed an increasing trend, particularly among pregnant and lactating women. Several studies reported emerging “modern” dietary patterns characterized by higher consumption of meat, fats, and processed foods, alongside limited dietary diversity. **Conclusions:** Overall, high-altitude populations exhibit distinctive macronutrient profiles shaped by environmental adaptation and nutritional transition, underscoring the need for altitude-specific dietary guidance and targeted public health interventions.

## 1. Introduction

### 1.1. Macronutrients and Human Health

A large amount of evidence indicates that dietary consumption patterns in different regions are closely related to the incidence of various chronic diseases (such as cardiovascular diseases, diabetes, and obesity) [1,2]. Macronutrients—carbohydrates, proteins, and fats—constitute the core components of dietary intake and play a central role in maintaining energy balance and metabolic health [3]. Accumulating evidence suggests that macronutrient intake patterns differ substantially across regions, populations, and lifestyles, and that such differences may influence nutritional status and long-term health outcomes [4,5]. An appropriate balance of macronutrient intake is believed to help reduce the risk of chronic diseases and promote overall health, while an unbalanced intake can lead to excessive or insufficient energy or nutrients, thereby causing malnutrition, metabolic disorders, and related diseases [6].

In the field of nutritional epidemiology, various dietary assessment tools—such as food frequency questionnaires (FFQ), 24 h dietary recall, and three-day weighed food records—are widely used to evaluate the dietary intake and nutritional composition of individuals or groups [7,8]. Data obtained from these instruments are typically used in two analytical approaches: (1) Dietary patterns are classified through statistical models, and (2) specific food and nutrient intakes are directly reported [9,10]. Such systematic assessments provide valuable insights into the potential associations between dietary behaviors and health outcomes.

### 1.2. Nutritional Characteristics and Research Significance of High-Altitude Populations

Regions located at altitudes above 1500 m are generally defined as high-altitude areas [11], and approximately 400 million people worldwide reside in such environments [12]. Living in high-altitude areas for a long time exposes the human body to a series of unique physiological challenges, such as decreased air pressure and oxygen partial pressure, low temperatures, and increased ultraviolet radiation [13,14]. At the same time, previous studies have shown that chronic exposure to hypoxia can induce oxidative stress, resulting in damage to biological macromolecules such as nucleic acids, lipids, and proteins. This increases the risk of tissue oxidative injury and promotes the development of altitude-related diseases [15,16,17]. Dietary nutrients play a key role in regulating redox balance, and variations in their intake can influence the degree of oxidative stress [18,19,20,21]. Certain dietary factors may act as pro-inflammatory or pro-oxidative agents within the body, disrupting redox balance and triggering adaptive activation of antioxidant defense systems to maintain cellular function [22].

Earlier evidence suggests that dietary patterns in high-altitude regions are strongly influenced by local agricultural systems and habitual food consumption, with some regions displaying carbohydrate-dominant diets and comparatively low protein intake [23]. In recent years, with the advancement of regional economies, transportation infrastructure, and market integration, the dietary structure of high-altitude populations has gradually diversified, reflecting a transition from traditional to modern dietary patterns [24].

Existing evidence on dietary structure and macronutrient intake among high-altitude populations remains fragmented, and comprehensive systematic reviews are scarce. Moreover, substantial heterogeneity exists across studies in terms of target populations, methodological approaches, and outcome measures. Therefore, this study aims to systematically synthesize available dietary intake data from high-altitude populations to characterize current patterns of energy and macronutrient intake, thereby providing an evidence base to support future nutritional interventions, policy development, and fundamental research in high-altitude settings.

## 2. Methods

### 2.1. Guidelines and Registration

**Figure 1:**
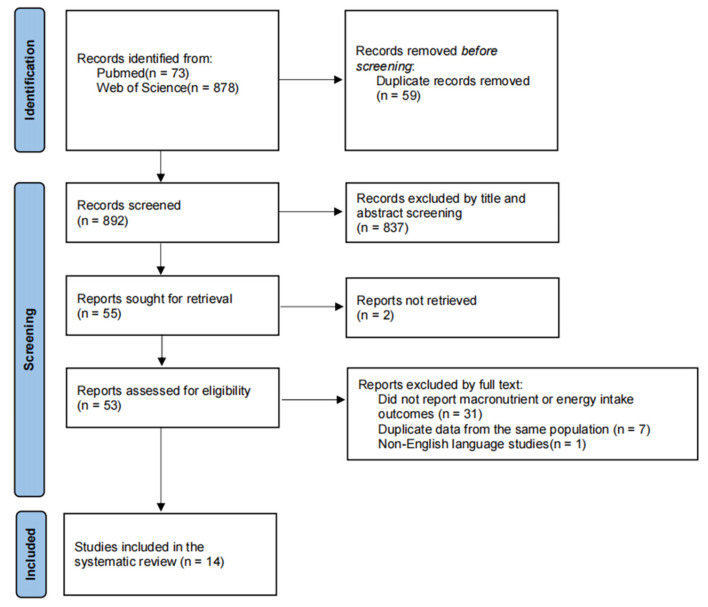
Flow diagram of the study selection process.

This systematic review was conducted based on existing evidence regarding macronutrient and energy intake among high-altitude populations and was performed in accordance with the Preferred Reporting Items for Systematic Reviews and Meta-Analyses (PRISMA) 2020 guidelines. The study selection process is presented in the PRISMA flow diagram (Figure 1), and the PRISMA 2020 checklist is provided in the Supplementary Materials. The review protocol was not prepared or registered.

### 2.2. Information Sources and Search Strategy

A comprehensive literature search was performed using the PubMed and Web of Science databases. According to the study objectives, three main keyword domains were considered:

1. Studies focusing on high-altitude populations;
2. Macronutrient and energy intake or metabolism;
3. Dietary patterns or nutritional characteristics.

Boolean search strategies were constructed by combining these terms, as shown in Table 1, to retrieve relevant literature published between 1990 and 2025. The search aimed to identify all available studies related to macronutrient and energy intake among populations residing in high-altitude regions. The same final Boolean search string (Table 1) was applied to both PubMed and Web of Science. No database-specific filters were used, including restrictions on language, publication date, study design, or population characteristics. All searches were conducted without limitations to maximize sensitivity and ensure the comprehensive identification of studies relevant to the research topic. The literature search was last updated in March 2025.

### 2.3. Inclusion and Exclusion Criteria

Inclusion and exclusion criteria were formulated according to the PICOS framework—Participants, Interventions, Comparators, Outcomes and Study design. The detailed criteria are summarized in Table 2. Studies were eligible for inclusion if they met the following criteria: (1) Population: human populations residing at high altitude (≥1500 m); (2) Exposure/Intervention: assessment of habitual dietary intake using validated dietary assessment methods (e.g., FFQ, 24 h dietary recall, dietary records, or structured dietary interviews); (3) Comparison: comparisons across altitude levels, population subgroups, or relative to dietary reference intakes (DRIs); (4) Outcomes: quantitative or semi-quantitative data on energy intake, macronutrient intake, or dietary patterns; (5) Study design: observational studies, including cross-sectional, cohort, case–control, and ecological studies. Studies were excluded if they were animal or in vitro studies, reviews or conference abstracts, lacked dietary intake data, reported duplicate populations, or failed to provide sufficient methodological detail.

### 2.4. Study Selection

As shown in Figure 1, based on the predefined search strategy, a total of 73 and 878 records were identified through systematic searches of the PubMed and Web of Science databases, respectively. After removing 59 duplicate records, 892 records were retained for screening. Title and abstract screening was conducted independently by a single reviewer (Yiyan Huang), during which 837 records were excluded for irrelevance to the research focus, leaving 55 reports sought for retrieval. Among these, 2 reports could not be retrieved, and 53 full-text articles were assessed for eligibility. Full-text assessment was independently performed by two authors (Yiyan Huang and Bin Li) in accordance with the predefined inclusion and exclusion criteria, with a specific focus on macronutrient intake and dietary structure among high-altitude populations. During the full-text review, 39 articles were excluded, including 31 studies that did not report macronutrient or energy intake outcomes, 7 studies reporting duplicate data from the same population, and 1 non-English language study. Following this process, 14 studies were ultimately included in the systematic review for data extraction and qualitative synthesis. Any disagreements during the screening and eligibility assessment were resolved through discussion between the two authors.

### 2.5. Data Extraction

Based on the research objectives, relevant information was independently extracted from all eligible studies by 2 reviewers. Any discrepancies in data extraction were resolved through discussion, and if consensus could not be reached, a third author was consulted for final decision-making. The extracted data primarily included the following aspects:

1. Author(s) and year of publication;
2. Study objectives;
3. Study design and methodology;
4. Study population;
5. Macronutrient intake levels or dietary structure characteristics;
6. Nutritional status of the study population.

The primary outcomes of interest were macronutrient intake levels (energy, carbohydrate, protein, and fat) and dietary structure or dietary pattern characteristics. Secondary outcomes included indicators of nutritional status and altitude-related contextual information of the study populations.

### 2.6. Altitude Definition and Classification

When the residential altitude was explicitly reported, the stated altitude was directly extracted. High-altitude exposure was defined as a residential altitude of ≥1500 m. For structured interpretation and comparability, studies were further categorized into three altitude bands in line with commonly used classifications in high-altitude and public health research: high altitude (1500–2500 m), very high altitude (2500–3500 m), and extremely high altitude (≥3500 m) [25]. When exact residential altitude was not reported, altitude classification was inferred based on established elevation information of the study region or administrative unit described in the original publication. For studies conducted across multiple regions, the altitude of the region included in the primary analysis was used for classification.

### 2.7. DRI Comparison and Operational Classification

For studies reporting quantitative dietary intake (total energy in kcal/day and macronutrients in g/day), macronutrient energy contributions were calculated using Atwater conversion factors (carbohydrate 4 kcal/g; protein 4 kcal/g; fat 9 kcal/g). Specifically, %E was calculated as:

$$\%E_{CHO}=\frac{4\times CHO\left(g\right)}{Energy\left(kcal\right)}\times 100$$

$$\%E_{Protein}=\frac{4\times Protein\left(g\right)}{Energy\left(kcal\right)}\times 100$$

$$\%E_{Fat}=\frac{9\times Fat(g)}{Energy(kcal)}\times 100$$

The derived %E values were then classified relative to the Acceptable Macronutrient Distribution Range (AMDR) as below (<lower bound), within (between bounds), or above (>upper bound). For Chinese high-altitude adult populations, the AMDR defined in Chinese DRIs (WS/T 578.1—2017) was applied (CHO 50–65%E; fat 20–30%E; protein 10–15%E). For the child study conducted in the northern Argentinian highlands, international AMDR values for children/adolescents (IOM) were intended; however, this study did not report macronutrient grams, so AMDR classification could not be calculated. Studies reporting only energy/BMR or partial macronutrients were summarized descriptively and were not forced into AMDR categories.

### 2.8. Quality Assessment

Methodological quality assessment of the included studies was conducted using the Quality Assessment Tool for Studies with Diverse Designs (QATSDD) [26]. This tool was selected because the included studies were predominantly observational and encompassed multiple research designs, for which QATSDD enables consistent appraisal. Two authors (Yiyan Huang and Bin Li) independently assessed the methodological quality of each study, with each item scored on a scale from 0 to 3. Total QATSDD scores were expressed as a percentage of the maximum possible score. While a 50% threshold has been commonly used in previous applications, we adopted a more conservative cut-off of 60%, with studies scoring ≥60% considered to be of moderate to high methodological quality. When discrepancies between the two reviewers exceeded four points, a third author (Xiaodan Huang) was consulted to discuss and resolve disagreements until consensus was reached.

## 3. Results

### 3.1. Study Characteristics

After screening, a total of 14 studies were included in the final analysis. The majority of these studies employed a cross-sectional design [9,24,27,28,29,30,31,32,33,34], while 2 were cohort studies [35,36], 1 was an ecological study [37], and 1 was a case–control study [38]. The cross-sectional studies primarily aimed to describe dietary intake patterns among high-altitude populations, whereas the cohort studies pursued long-term objectives, such as exploring associations between dietary patterns and health outcomes or evaluating the effects of different dietary interventions on chronic disease risk. Regarding the study populations, most investigations focused on permanent residents of the Qinghai–Xizang Plateau in China [9,28,30,33]. 1 study examined populations from the northwestern highlands of Argentina [29], 2 studies involved populations residing along countries of the Yarlung Tsangpo River Basin [9,33], and 1 study investigated communities in the Indian Himalayas [38]. Most studies targeted healthy adult populations, with 2 studies involving children [29,38] and 2 focusing exclusively on female participants [28,35].Concerning sample size, the cross-sectional studies generally included fewer than 1000 participants, while the 2 cohort studies varied substantially in scale [32,36]. The case–control study involved approximately 2000 participants. Overall, there was considerable variation in sample size across different study designs, which may influence the representativeness and comparability of the findings. In terms of altitude distribution, 6 studies were conducted at extremely high altitude (≥3500 m) [24,27,28,31,32,35], 1 study at very high altitude (2500–3500 m) [30], and 4 studies were classified as mixed-altitude due to populations spanning multiple altitude categories [9,29,33,34]. 3 studies did not report explicit residential altitude or geographic information [36,37,38]. All quantitative studies reporting adult energy and macronutrient intake with defined altitude were conducted at extremely high altitude (≥3500 m). Methodological quality scores of included studies assessed using QATSDD are presented in Supplementary Table S1.

### 3.2. Dietary and Nutrient Intake Characteristics

Among the studies included in this systematic review, 6 articles specifically reported quantitative data on macronutrient intake among high-altitude populations (Table 3). Of these, 3 studies focused on special populations such as children or pregnant women [28,29,35], while the other 3 studies targeted the general adult population [24,31,37]. Another study investigated the differences in metabolic rates between men and women in high-altitude areas [27]. Among the studies providing quantitative nutrient data, 1 reported macronutrient intake levels using median values [28], whereas the other 5 used means and standard deviations to describe central tendencies [24,29,35,37].

### 3.3. Energy and Macronutrient Intake Characteristics

Regarding energy intake, substantial variability was observed among studies conducted in extremely high-altitude adult populations. The lowest mean daily energy intake was reported among adults in Naqu Prefecture [31], whereas the highest intake was observed among pregnant women [35]. In other studies, adult energy intake was generally concentrated within the range of approximately 2000–2200 kcal/day [24,28], indicating considerable heterogeneity in reported energy intake across studies.

With respect to macronutrient intake, pronounced population-specific differences were observed across high-altitude studies. Pregnant women residing in high-altitude areas consistently exhibited higher intakes of both protein and fat [35]. whereas adult residents of Naqu Prefecture showed comparatively lower intakes of these two macronutrients [31]. Among other high-altitude populations, protein intake generally fell within an intermediate range, without a clear monotonic pattern related to altitude alone [24,28]. Carbohydrate intake displayed a different pattern, with the highest intake was observed among mothers of children aged 0–2 years [28], whereas residents of the Nam Co region consistently showed the lowest carbohydrate intak [31]. In other adult populations living at high altitude, carbohydrate intake appeared relatively stable, clustering within a narrower range across studies [24].

### 3.4. Comparison with Recommended Dietary Intakes

By comparing the reported energy intake and macronutrient energy distribution across studies with the population-specific dietary reference intakes (Table 4), several consistent patterns emerged among high-altitude populations. Energy intake was generally within or close to recommended ranges, although suboptimal intake was observed in specific regions such as Naqu [31], whereas energy intake in all other study populations was within or above the recommended range [24,28,35], indicating that most high-altitude populations achieved adequate overall energy supply to meet physiological demands. In terms of macronutrient energy distribution, the proportion of energy derived from carbohydrates was lower than the recommended range exclusively in the Naqu population [31], while carbohydrate contribution in the remaining populations was generally within or exceeded the recommended range [24,28,35]. Among the four studies that reported fat-derived energy contribution, two demonstrated fat energy proportions above the recommended range, primarily among pregnant women and residents of certain high-altitude regions [31,35], suggesting a tendency toward higher fat contribution in specific subgroups. By contrast, protein energy contribution consistently fell within the recommended range across all studies included in the comparison [24,28,35], indicating a relatively stable and appropriate protein energy distribution among high-altitude populations.

### 3.5. Dietary Pattern Analysis

Among the included studies (Table 5), 4 did not provide quantitative descriptions of macronutrient intake but instead characterized the dietary patterns of high-altitude populations through statistical pattern classification analyses (Table 4). 3 studies reported notable age-related variations in dietary habits [9,30,32] while 1 focused on regional adaptation differences [36]. All 4 were published relatively recently, with the earliest in 2019. Notably, 3 of these studies focused primarily on Xizang populations living on the Qinghai–Xizang Plateau [9,30,32], whereas only 1 analyzed inter-regional dietary variation across western China [36].

From the summarized findings, several major dietary patterns among Xizang Plateau residents were identified: Modern dietary pattern: Characterized by high intake of meat (particularly poultry and pork), processed meat, sugar-sweetened beverages, salty snacks, oils, and fresh fruits. This pattern was observed among urbanizing herders and residents of the Sichuan Basin and was positively associated with obesity risk [30,32].

Traditional staple-based pattern: Defined by predominant consumption of staple grains (e.g., hulless barley and cereals), fruits, and vegetables [9]. It represents the most fundamental and historically stable diet of high-altitude regions. Similarly to the “urban” or “mixed staple” patterns reported by Peng et al. (2019) [30], which emphasizes vegetables, tubers, and refined carbohydrates but includes more external dietary elements.

High-protein pattern: Marked by greater consumption of eggs, meats (especially beef and mutton), and dairy products such as butter and yogurt [30]. Interestingly, Peng et al. (2019) [30] reported both “high” and “low” subtypes of this pattern, implying notable intra-group or definitional variability.

**Table 5:** Dietary Pattern Results.

Author (Year)	Sample	Main Findings: Dietary Patterns	Nutritional Status
Kong, 2022 [9]	Permanent residents (*n* = 617)	Three patterns: (1) Staple–fruit–vegetable; (2) Staple–meat–dairy; (3) Staple-only (dominant).	Not reported
Li, 2023 [32]	Xizang herders, Qinghai (*n* = 1913)	Modern, Urban, and Pastoral patterns; urbanized, educated groups favor modern diets; high adherence linked to obesity.	The prevalence of central obesity was 51.3% in men and 57.4% in women.
Lu, 2023 [36]	Multi-ethnic 18–79 yrs (*n* = 81,433)	Regional patterns: Sichuan (fish, dairy, produce high); Yunnan–Guizhou (animal oil, salt high); Qinghai–Tibet (coarse grains, tea).	Participants with dietary patterns more closely aligned with the Qinghai–Xizang Plateau diet had a higher mean BMI (24.6 ± 3.6 kg/m^2^).
Peng et al., 2019 [30]	Adults 18–84 yrs (*n* = 782)	Urban, Western, and Pastoral patterns; 93% consume beef/mutton daily; Western pattern younger demographic.	The prevalence of overweight and obesity was 58.4% and 26.6%, respectively, with the overall proportion of central obesity approaching 60%.

### 3.6. Dietary Characteristics Without Quantitative Macronutrient Data

Among the included studies (Table 6), three did not report quantitative macronutrient or energy intake but provided descriptive information on dietary structure and food consumption among high-altitude populations. All were cross-sectional studies involving different population groups, including high-altitude school-aged children in Himachal Pradesh, India, and Xizang residents and adults living along the Yarlung Tsangpo River [33,34,38]. Among school-aged children, consumption of animal-source foods and dairy products was relatively infrequent, accompanied by generally low vegetable intake [38]. Similarly, among adult residents living along the Yarlung Tsangpo River, dietary intake was highly staple-centered, with widespread daily consumption of traditional grain-based foods, reflecting a traditional grain-based dietary pattern [34]. Studies of adult Xizang populations further reported insufficient intake of most food groups, except for relatively high consumption of meat and soy products [33].

### 3.7. Energy Metabolism

Data on energy metabolism, assessed using basal metabolic rate (BMR), were reported in two studies (Table 7). Among adult Xizang nomads, Beall et al. reported mean BMR values of 1360 ± 190 kcal/day in men and 1239 ± 142 kcal/day in women [27]. In a pediatric population, Rossi et al. reported a mean BMR of 1145 ± 172 kcal/day among children aged 6–12 years residing in the northern Argentinian highlands [29].

### 3.8. Nutritional Status

Several included studies reported nutritional status indicators of high-altitude populations, including BMI, weight status categories, and central obesity, revealing substantial variation across population groups. Among children residing in high-altitude settings, both undernutrition and overnutrition were observed, with evidence of low body weight, stunting, and excess weight coexisting within the same populations [29]. Adult female and maternal populations generally exhibited BMI values within the normal range, including rural mothers and pregnant women, although BMI increased during pregnancy and the early postpartum period [28,35]. I Notably, some adult high-altitude populations demonstrated a high prevalence of excess body weight and central obesity, particularly in studies conducted among more urbanized or diet-transitioning groups [30,32,36]. Central obesity was consistently reported at relatively high levels in both men and women, even when mean BMI values fell within the overweight range [32,36]. Similarly, studies from Naqu Prefecture reported mean BMI values in the overweight range for both cases and controls, suggesting a substantial burden of excess body weight in this setting [31].

## 4. Discussion

This systematic review synthesized evidence from 14 studies published between 1996 and 2023 that examined energy and macronutrient intake, as well as dietary pattern characteristics, among high-altitude populations across the Qinghai–Xizang Plateau, Himachal Pradesh (India), and the Argentine Andes. The results reveal distinct regional and metabolic features in high-altitude diets. Overall, high-altitude diets were characterized by energy intake that was generally close to recommended levels, a relatively high contribution of carbohydrates to total energy intake, largely adequate protein intake, and a tendency toward increased fat consumption in certain populations. Together, these findings suggest that dietary intake among high-altitude populations reflects both long-standing dietary structures and emerging nutritional transitions, which may have implications for metabolic health in these environments.

### 4.1. Energy Intake and Metabolic Differences

Across studies, energy intake among high-altitude populations was generally close to, or slightly exceeded, recommended intake levels for the corresponding populations. Notable differences were observed between population groups, potentially reflecting variation in labor demands, local climatic conditions, and socioeconomic factors [39,40,41]. While high-altitude hypoxic and cold environments are generally associated with increased BMR, insufficient energy intake in some regions may prompt compensatory metabolic adjustments [42]. In line with this, Beall et al. (1996) observed lower BMR values among Xizang nomads during periods of reduced energy intake [27]. Although compensatory, chronic energy shortfall may impair muscle growth, immune, and reproductive functions [43,44].

### 4.2. Macronutrient Intake Structure and Trends

In the present review, dietary patterns among high-altitude populations remain predominantly carbohydrate-based, while protein intake is generally adequate across population groups. However, fat intake approaches the upper recommended limit and reaches relatively high levels in certain subgroups. Notably, with increasing altitude, the macronutrient profile appears to shift toward a greater contribution from dietary fat, suggesting a transition from a traditionally high-carbohydrate diet to a more mixed, energy-dense dietary pattern [24,31]. This shift may be partly related to metabolic characteristics associated with high-altitude environments, and potential sex-specific differences in substrate utilization may further modulate this pattern. Research conducted at an altitude of approximately 4300 m indicates that the fat oxidation rate of females is higher than the consumption of carbohydrates [45]. This phenomenon of metabolic adaptation for different genders reflects the need to formulate customized dietary recommendations based on the population rather than a uniform energy standard. This adaptation parallels observed increases in dietary fat and protein intake among high-altitude women. However, if high-fat diets surpass metabolic capacity, they may raise obesity and cardiovascular risk [46,47]. Li et al. (2023) found significantly higher obesity prevalence among pastoralists adhering to modern high-fat/high-sugar patterns [32].

### 4.3. Comparison of Macronutrient Intake Between High-Altitude and Lowland Populations

Compared with nationally representative data from lowland populations, macronutrient intake among high-altitude populations does not exhibit a uniform upward shift. For instance, the 2010–2012 China National Nutrition and Health Survey reported a mean daily energy intake of approximately 2163 kcal, with average protein and fat intakes of about 64 g/d and 80 g/d, respectively [48]. In contrast, substantial heterogeneity is also evident within high-altitude regions themselves. For example, diets on the Andean Plateau tend to be more staple-based and relatively lower in fat intake, suggesting that there is no single, unified pattern of macronutrient adaptation associated with high-altitude living [49]. Taken together, the findings of the present review suggest that a defining feature of high-altitude populations is not uniformly elevated intake levels, but rather pronounced variability in macronutrient consumption across regions and population subgroups. In some extremely high-altitude settings, energy and protein intakes appear to be comparatively low [31], whereas specific groups—such as pregnant women—may exhibit markedly higher intakes of fat and protein [35]. Therefore, dietary recommendations and nutritional interventions for high-altitude populations should move beyond altitude gradients alone and also account for dominant food sources, market accessibility, seasonal food availability, and population characteristics, in order to enhance both feasibility and contextual relevance.

### 4.4. Staple-Food Structure and Dietary Diversity

Hulless barley (tsampa) remains the most representative staple of Xizang diets—consumed daily by nearly all rural residents [50]. While this ensures adequate energy intake, it also limits nutritional variety, particularly micronutrients such as selenium, which is linked to Kashin–Beck disease [51]. Recent surveys show that although barley is still the main energy source, the intake of rice and wheat has increased, indicating gradual diversification [9]. However, vegetable and fruit consumption remains low [24], restricting vitamin, mineral, and fiber intake and weakening antioxidant and chronic disease protection [52]. Lifestyle and education levels also influence dietary quality [53]; higher education correlates with more balanced diets and healthier behaviors [50]. Therefore, nutritional interventions in high-altitude regions should integrate dietary education with health awareness programs to improve overall diet quality. In addition to ensuring adequate intake, particular attention should be given to enhancing dietary diversity among high-altitude populations.

### 4.5. Dietary Patterns and Population Differences

Available evidence indicates that dietary patterns in high-altitude regions are shifting from relatively homogeneous structures toward greater diversity, with widening differences across population groups. Existing studies suggest that this transition is reflected both in the diversification of traditional staple-based diets and in urbanization-related changes, including increased consumption of processed foods, meat, and sugar-sweetened beverages alongside a relative decline in traditional staple foods [9,32]. This “nutritional transformation” has raised concerns about energy excess and the risk of non-communicable diseases [46,54,55]. Regional differences still exist: Lu et al. (2023) reported that residents of the Qinghai–Xizang Plateau consume more coarse grains and tea, but they consume less animal fat and alcohol than residents in low-altitude areas [36]. These patterns suggest that dietary structures in high-altitude populations are currently in a transitional phase shaped by both traditional practices and modern influences. Accordingly, targeted strategies may be warranted to promote dietary diversity and nutritional balance, while minimizing the risk of excessive energy intake and related metabolic disorders.

This systematic review has several limitations. Most of the included studies relied on food frequency questionnaires (FFQs) or 24 h dietary recalls (24HDRs), both of which are subject to recall bias and measurement error. FFQs require participants to estimate average dietary intake over extended periods, a process that may be particularly challenging in high-altitude settings due to seasonal variation in food availability and differences in educational background among participants [56,57]. In contrast, 24HDRs capture short-term dietary intake but may be less representative of habitual or long-term dietary patterns, especially in populations experiencing substantial day-to-day variation in food supply, daily activities, or energy expenditure [58,59]. These methodological limitations may result in the over- or underestimation of specific food groups, thereby affecting the identification and interpretation of dietary patterns [60]. In addition, owing to substantial heterogeneity among the included studies in terms of study design, population characteristics, dietary assessment methods, and outcome definitions, a formal meta-analysis was not conducted. Instead, the findings were synthesized narratively, which precluded the generation of pooled effect estimates and limited formal exploration of between-study variability. Consequently, traditional sensitivity analyses and formal statistical assessments of publication bias were not applicable [61]. What is more, the overall number of studies investigating dietary intake among high-altitude populations remains limited, and the evidence base for specific population groups is relatively sparse, which restricts the assessment of the generalizability of observed dietary structures. Furthermore, the available evidence shows a marked geographic concentration, with most studies conducted in the Qinghai–Xizang Plateau and along the Yarlung Tsangpo River basin. This uneven geographic distribution may further limit the extrapolation of the findings to high-altitude populations in other regions with different ecological, cultural, and dietary contexts.

## 5. Conclusions

This systematic review demonstrates that high-altitude populations exhibit distinctive macronutrient intake characteristics shaped by environmental adaptation and sociocultural transition. The overall situation of dietary diversity is improving, but imbalances in macronutrient intake remain common—excess intake of fat and protein and inadequate intake of fruits, vegetables, and micronutrients. The existing nutrient intake guidelines are mainly based on the low-altitude population and lack the consideration of altitude-related metabolic changes, such as the increase in basal energy expenditure and changes in fat oxidation.

Future research should incorporate multiple dimensions of factors (including gender, age, occupation, and altitude gradient) into the dietary assessment framework altitude levels. This will provide a more solid foundation for setting nutritional reference values at different altitudes. At the same time, the public health policy should not only focus on dietary guidance, but also include measures such as food supply, nutrition education, and community monitoring. The establishment of a nutrition policy for the plateau population with a scientific basis is not only beneficial for the health of the plateau population, but also important for further exploring the metabolic adaptation characteristics of the human body to the high-altitude environment.

## Figures and Tables

**Table 1:** Construction of Search Strategy.

Concept	Search Terms
High-altitude populations	“Tibetan Plateau” OR “Qinghai-Tibet Plateau” OR “high-altitude” OR “Tibetans” OR “Energy Expenditure” OR “Caloric Metabolism”
Macronutrient and energy intake	“nutrition” OR “nutrient intake” OR “macronutrient intake” OR “energy intake” OR “protein” OR “carbohydrate” OR “fat” OR “macronutrient” OR “caloric intake” OR “energy consumption”
Dietary and nutritional aspects	“dietary intake” OR “nutritional status” OR “dietary patterns”
Final Boolean search string	(“Tibetan Plateau” OR “Qinghai-Tibet Plateau” OR “Tibet” OR “high-altitude regions” OR “Tibetan”) AND (“nutrition” OR “nutrient intake” OR “macronutrient intake” OR “energy intake” OR “protein” OR “carbohydrate” OR “fat” OR “macronutrient” OR “caloric intake” OR “energy consumption”) AND (“dietary intake” OR “nutritional status” OR “dietary patterns”)

**Table 2:** PICOS-based inclusion and exclusion criteria.

Population (P)	Intervention (I)	Comparison (C)	Outcomes (O)	Study Design (S)
Residents living at altitudes ≥1500 m;	Habitual dietary intake assessment; FFQ; 24 h dietary recall; Dietary records; Structured interviews	Different altitude levels; Population subgroups; Comparison with DRIs	Energy intake and metabolism; Macronutrient intake; Dietary patterns or structure	Cross-sectional; Cohort; Case–control; Ecological studies

**Table 3:** Characteristics of Studies Included in the Systematic Review.

Author (Year)	Study Objective	Study Design	Population/Sample (*n*)	Residential Altitude (m)
Rossi et al., 2018 [29]	Evaluate diet and polyphenol intake in Argentine highland schoolchildren.	Cross-sectional study	Children 6–12 years, *n* = 241	1500–3700
L. Wang et al., 2021 [24]	Examine food structure vs. environmental factors in Qinghai–Xizang Plateau.	Ecological study	General residents	>4000
Z. Wang et al., 2010 [28]	Describe diets of Xizang rural mothers and nutrient intake.	Cross-sectional study	Mothers of 0–2 yr children, *n* = 386	3685
Dao et al., 2023 [35]	Analyze maternal nutrition and lactation in traditional high-altitude areas.	Cross-sectional study	Pregnant women (24–30 yr), Hongyuan, *n* = 62	≈3600
Ge et al., 1997 [37]	Compare dietary habits among 20 ethnic groups in China.	Cross-sectional study	Tibetan subgroup within *n* = 9304 total	N/A
Beall et al., 1996 [27]	Examine BMR seasonal variation in Phala nomads at high altitude.	Cohort study	Adult nomads, Tibet, *n* = 52	4850–5450
Cui, 2022 [31]	Study associations between diet, Xizang foods, and high-altitude polycythemia (HAPC).	Case–control	Naqu residents (*n* = 1171)	>4500
Kong, 2022 [9]	Analyze major dietary patterns and regional variation on Qinghai–Xizang Plateau.	Cross-sectional	Permanent residents (*n* = 617)	≈3000–5000
Li, 2023 [32]	Examine links between diet and obesity among Xizang herders.	Cohort	Xizang herders, Qinghai (*n* = 1913)	>4000
Lu, 2023 [36]	Assess links between diet and blood pressure in SW China.	Cohort	Multi-ethnic 18–79 yrs (*n* = 81,433)	N/A
Peng et al., 2019 [30]	Examine dietary patterns and blood pressure in urbanized Xizang pastoralists.	Cross-sectional	Adults 18–84 yrs (*n* = 782)	2800
Gupta et al., 2017 [38]	Assess vitamin B12 and folate deficiency among high-altitude children in Himachal Pradesh, India.	Cross-sectional study	School-aged children (6–18 years), *n* = 215	N/A
Jia et al., 2023 [34]	Determine Se and Zn intake in staple foods along Yarlung Tsangpo River.	Cross-sectional/ecological	Xizang residents, *n* = 244	≈3000–4500
Zhou et al., 2021 [33]	Explore dietary habits of Xizang adults for targeted nutrition interventions.	Cross-sectional study	Adults along Yarlung Tsangpo River, *n* = 552	≈3000–4500

**Table 4:** Summary of reported macronutrient and energy intake among high-altitude populations.

Author (Year)	Population/Sample (*n*)	Nutrient or Energy Intake	Relative to DRIs *	Nutritional Status
Rossi et al., 2018 [29]	Children 6–12 years, *n* = 241	Energy 1547 ± 478 kcal/day;	Energy ✓; CHO —; Protein —; Fat —	Underweight 2.2%; Low weight 12.7%; Overweight 12.7%; Obesity 7.4%; Stunting 4.8%
L. Wang et al., 2021 [24]	General residents	Energy 2156 kcal/day; CHO 316 g/day; Protein 73.5 g/day; Fat 66.5 g/day.	Energy ✓; CHO ✓; Protein ✓; Fat ✓	Not reported
Z. Wang et al., 2010 [28]	Mothers of 0–2 year children, *n* = 386	Energy 2097 kcal/day; CHO 357 g/day; Protein 58 g/day; Fat 57 g/day.	Energy ✓; CHO ↑; Protein ✓; Fat ✓	BMI < 18.5 kg/m^2^: 10.3%; BMI 18.5–24.9 kg/m^2^: 81.4%; BMI 25–29.9 kg/m^2^: 8.3%
Dao et al., 2023 [35]	Pregnant women (24–30 yr), Hongyuan, *n* = 62	Energy 2487 kcal/day; Fat 124 g/day; Protein 92.3 g/day; CHO 311 g/day.	Energy ✓; CHO ✓; Protein ✓; Fat ↑	Pre-pregnancy BMI 22.1 ± 3.3 kg/m^2^; 1-month postpartum BMI 24.4 ± 2.7 kg/m^2^
Ge et al., 1997 [37]	Xizang subgroup within *n* = 9304 total	Men: 3262 kcal/day, 98 g protein; Women: 2979 kcal/day, 85.5 g protein.	Energy ↑; CHO —; Protein ✓; Fat —	Not reported
Cui, 2022 [31]	Naqu residents (*n* = 1171)	Energy: 1689.00 ± 839.25 kcal/day Protein: 48.11 ± 34.16 g/day Carbohydrate: 203.94 ± 120.32 g/day Fat: 76.14 ± 37.30 g/day	Energy ↓; CHO ↓; Protein ✓; Fat ↑	Cases: 26.80 ± 5.04 kg/m^2^; Controls: 26.49 ± 6.51 kg/m^2^

*: ✓ within reference range; ↑ above reference range; ↓ below reference range; — not reported/not assessable.

**Table 6:** Descriptive dietary characteristics.

Author (Year)	Sample	Main Findings: Dietary Patterns	Nutritional Status
Gupta et al., 2017 [38]	School-aged children (6–18 years), *n* = 215	62% consumed animal products weekly; 50% consumed dairy daily; low vegetable intake.	Not reported
Jia et al., 2023 [34]	Xizang residents, *n* = 244	99.6% consumed tsampa daily; 53.7% wheat, 72.5% rice daily.	Not reported
Zhou et al., 2021 [33]	Adults along Yarlung Tsangpo River, *n* = 552	Low intake of most foods except excess meat and soy.	Not reported

**Table 7:** Basal Metabolic Rate (BMR) among High-Altitude Populations.

Author (Year)	Population/Sample (*n*)	Basal Metabolic Rate (BMR)
Beall et al., 1996 [27]	Adult nomads, Tibet, *n* = 52	Men: 1360 ± 190 kcal/day; Women: 1239 ± 142 kcal/day.
Rossi et al., 2018 [29]	Children 6–12 years, *n* = 241	BMR 1145 ± 172 kcal/day.

## Data Availability

Data extracted and analyzed during this systematic review are derived from published studies and are presented within the article and its Supplementary Materials. No new datasets or analytic code were generated for this review.

## Supplementary Materials

The following supporting information can be downloaded at: https://www.mdpi.com/article/10.3390/nu18040572/s1, Table S1: QATSDD Summary, Table S2: QATSDD Supplementary. PRISMA_2020_checklist. Reference [62] is cited in the Supplementary Materials.

## Abbreviations

The following abbreviations are used in this manuscript:

BMR	Basal Metabolic Rate
CHO	Carbohydrate
CONSORT	Consolidated Standards of Reporting Trials
DRI	Dietary Reference Intake
FFQ	Food Frequency Questionnaire
HAPC	High-Altitude Polycythemia
PRISMA	Preferred Reporting Items for Systematic Reviews and Meta-Analyses
RCT	Randomized Controlled Trial
STROBE	Strengthening the Reporting of Observational Studies in Epidemiology
24HDR	24-Hour Dietary Recall
