# Supplementary material for: Current Status of Macronutrient and Energy Intake and Metabolism Among High-Altitude Populations: A Systematic Review
Source: Nutrients. 2026 Feb 9;18(4):572. doi: 10.3390/nu18040572 (PMC12943662; doi:10.3390/nu18040572)
Supplement: Supplementary file 1 [file nutrients-18-00572-s001.zip › Table S1 QATSDD Summary.pdf]

Table S1 QATSDD Summary

First author	Rossi et al.	Wang et al.	Wang et al.	Dao et al.	Ge et al.	Beall et al.	Gupta et al.
Year	2017	2021	2010	2023	1997	1996	2017
Design	Cross-sectional	Ecological	Cross-sectional	Cross-sectional	Ecological	Cohort	Cross-sectional
C1	1	2	2	2	1	3	1
C2	3	3	3	3	3	3	3
C3	3	3	3	3	3	3	3
C4	3	2	3	1	3	2	3
C5	3	3	3	2	3	3	2
C6	3	3	3	3	3	3	3
C7	2	3	3	3	2	3	2
C8	2	1	3	2	3	3	3
C9	1	2	2	2	2	3	2
C10	3	3	3	3	3	3	3
C11	N/A	N/A	N/A	N/A	N/A	N/A	N/A
C12	2	3	3	2	2	3	2
C13	2	3	3	2	2	3	2
C14	N/A	N/A	N/A	N/A	N/A	N/A	N/A
C15	1	0	2	1	0	2	0
C16	2	2	3	2	3	3	2
Total (applicable)	31	33	39	31	33	40	31
Max	42	42	42	42	42	42	42
Percent	73.8%	78.6%	92.9%	73.8%	78.6%	95.2%	73.8%

First author	Cui et al.	Kong et al.	Li et al.	Lu et al.	Peng et al.	Jia et al.	Zhou et al.
Year	2022	2022	2023	2023	2019	2023	2021
Design	Case–control	Cross-sectional	Cohort	Cross-sectional	Cross-sectional	Cross-sectional	Cross-sectional
C1	2	2	2	3	2	2	1
C2	3	3	3	3	3	3	3
C3	3	3	3	3	3	3	3
C4	2	2	2	3	2	1	1
C5	3	3	3	3	3	2	2
C6	3	3	3	3	3	3	3
C7	2	2	2	3	2	3	3
C8	3	3	3	3	3	2	1
C9	2	2	2	3	1	3	2
C10	3	3	3	3	3	3	3
C11	N/A	N/A	N/A	N/A	N/A	N/A	N/A

C12	3	3	3	3	3	3	3
C13	3	3	3	3	2	3	3
C14	N/A	N/A	N/A	N/A	N/A	N/A	N/A
C15	1	1	1	1	1	0	2
C16	3	3	3	3	3	1	3
Total (applicable)	36	36	36	40	34	32	33
Max	42	42	42	42	42	42	42
Percent	85.7%	85.7%	85.7%	95.2%	81%	76.2%	78.6%
