# Supplementary material for: Current Status of Macronutrient and Energy Intake and Metabolism Among High-Altitude Populations: A Systematic Review
Source: Nutrients. 2026 Feb 9;18(4):572. doi: 10.3390/nu18040572 (PMC12943662; doi:10.3390/nu18040572)
Supplement: Supplementary file 1 [file nutrients-18-00572-s001.zip › Table S2 QATSDD Supplementary.pdf]

Table S2 QATSDD Supplementary

Item No.	QATSDD Criterion	Score (0–3)
1	Explicit theoretical framework	
2	Statement of aims and objectives	
3	Clear description of research setting	
4	Evidence of sample size consideration / justification	
5	Representative sample of target population	
6	Description of data collection procedure	
7	Rationale for choice of data collection tool(s)	
8	Reliability of measurement tools (quantitative only)	
9	Validity of measurement tools (quantitative only)	
10	Fit between research question and data analysis method	
11	Assessment of analytical rigor	
12	User / participant involvement in study design	
13	Strengths and limitations critically discussed	
14	Evidence of ethical considerations	
15	Evidence of reflexivity (qualitative only)	
16	Conclusions supported by the results	

Quality of included studies was assessed using the Quality Assessment Tool for Studies with Diverse Designs (QATSDD). Each item was scored on a 4-point Likert scale (0–3), where 0 = not at all, 1 = very slightly, 2 = moderately, and 3 = complete. Total scores were calculated separately according to study design.
